# The auditory brain in action: Intention determines predictive processing in the auditory system—A review of current paradigms and findings

**DOI:** 10.3758/s13423-021-01992-z

**Published:** 2021-09-10

**Authors:** Betina Korka, Andreas Widmann, Florian Waszak, Álvaro Darriba, Erich Schröger

**Affiliations:** 1 Institute of Psychology – Wilhelm Wundt, Leipzig University, D-04109 Leipzig, Germany; 2 Leibniz Institute for Neurobiology, D-39118 Magdeburg, Germany; 3 Department of Neurology, Otto-von-Guericke University, D-39120 Magdeburg, Germany; 4 Université de Paris, CNRS, Integrative Neuroscience and Cognition Center, F-75006 Paris, France

**Keywords:** Intentional action, Action–effect predictions, Auditory processing, Ideomotor theory, Predictive coding, Extended auditory event representation system

## Abstract

According to the ideomotor theory, action may serve to produce desired sensory outcomes. Perception has been widely described in terms of sensory predictions arising due to top-down input from higher order cortical areas. Here, we demonstrate that the action intention results in reliable top-down predictions that modulate the auditory brain responses. We bring together several lines of research, including sensory attenuation, active oddball, and action-related omission studies: Together, the results suggest that the intention-based predictions modulate several steps in the sound processing hierarchy, from preattentive to evaluation-related processes, also when controlling for additional prediction sources (i.e., sound regularity). We propose an integrative theoretical framework—the extended auditory event representation system (AERS), a model compatible with the ideomotor theory, theory of event coding, and predictive coding. Initially introduced to describe regularity-based auditory predictions, we argue that the extended AERS explains the effects of action intention on auditory processing while additionally allowing studying the differences and commonalities between intention- and regularity-based predictions—we thus believe that this framework could guide future research on action and perception.

Two recent developments are of particular interest for the current review. First, for the longest time, research on human action focused on stimulus–response behaviour, where acting was merely considered a response to environmental demands. The past few decades, however, marked a shift, in that researchers’ interest in actions that serve to produce desired effects in the environment increased progressively. Second, the way we understand perception has come a long way, too—the general agreement being that perception is not an entirely stimulus-driven process, but that it is an interplay between bottom-up processing and top-down expectations.

The bottom-up vs. top-down distinction has been put forward most prominently by the predictive coding theory of perception (Friston, 2005, 2010; Mumford, 1992; Rao & Ballard, 1999). According to this theory, the brain is constantly predicting future events by accumulating evidence via integrated feed-forward and feed-backward loops. First, predictions concerning the expected input propagate from higher to lower brain areas, where a comparison against the actual sensory input is carried out. The prediction error resulting from this comparison is then carried back up the cortical hierarchy, the goal being to minimize prediction error and thus improve processing efficiency (Feldman & Friston, 2010).

Information processing in the auditory system has also been widely described in terms of predictive top-down processes that can be derived from several sources: general regularities in the environment, visual–auditory associations, long-term learning (such as in language, for example), and/or our own actions (for comprehensive reviews, see Bendixen et al., 2012; Schröger et al., 2015). A number of reviews have assessed, for instance, the mechanisms underlying regularity-based predictions in oddball paradigms (Garrido et al., 2009; Näätänen et al., 2007) and their corresponding computational models (Schröger et al., 2014), sensory attenuation effects in self-generation studies (Horváth, 2015; Hughes et al., 2013a), or the interactive effects of attention and prediction (Lange, 2013; Schröger et al., 2015).

Here, we focus on the role of intentional action and action–effect predictions in the context of auditory processing. This review aims to fill a gap in the body of literature and demonstrate that intentional action represents a main source of top-down prediction in the auditory system. In the following sections, we first briefly describe the notion of intentional action in broader terms, as it has been formalized in the context of the ideomotor theory. Then, we review several lines of research, from sensory attenuation studies, to studies investigating mismatching and omissions of expected action effects. Finally, we integrate the findings of this research into a common perspective by proposing that the auditory event representation system (AERS), a model initially introduced to describe auditory regularity-based predictions (Schröger et al., 2014; Winkler & Schröger, 2015), can be extended to explain the effects on action intention and action–effect predictions on the auditory processing*.*

## The ideomotor framework of intentional action

Experience teaches us that certain actions have specific sensory effects. Once such action-effect couplings have been learned, we select our actions according to their desired consequences. This idea is at the core of the ideomotor theory of action control, which has been widely used to describe the mechanisms of intentional action (Greenwald, 1970; Hommel, 2003, 2013). Elsner & Hommel (2001) provided evidence supporting the ideomotor theory through a series of experiments in which they demonstrated that action–effect associations (key presses and tones, respectively) learned during an acquisition phase consistently modulated response selection and response speed in a subsequent test phase. This series of experiments demonstrates that action–effect associations are represented in a bidirectional way, an idea further elaborated by the theory of event coding (TEC), which postulates that perception and action operate on the same codes, event files, consisting of integrated sensorimotor networks (Hommel et al., 2001) that are retrieved in a very selective manner according to the current goals of the individual (Hommel, 2019).

Intention-driven action has been contrasted with stimulus-driven action (Herwig et al., 2007; Herwig & Waszak, 2009; Waszak et al., 2005). While the former serves goal-directed behaviour, the second is reflexive in nature, the actions being selected according to the stimulus. The two types of action presumably reflect different types of learning as well. In this regard, Herwig and colleagues showed in a series of behavioural experiments that reliable action-effect/ideomotor learning occurs only if in the acquisition phase participants can freely choose which action to perform (intention driven), but not if the action to be performed is cued (stimulus driven; Herwig et al., 2007; however, for counterevidence, see Janczyk et al., 2012; Pfister et al., 2011). These findings added to previous results suggesting that action-effect acquisition relies on some degree of intentionality (Waszak et al., 2005), as intentional but not the stimulus-driven action led to the elicitation of a readiness potential-like response in the EEG, a component that had previously been related to the generative process of intentional actions (Jahanshahi et al., 1995). However, contrasting views suggest that the acquisition of action–effect associations may be spontaneous rather than intentional (Hommel, 2013). Regardless of whether action effects are acquired intentionally or spontaneously, the agreement is that once the appropriate actions need to be selected in order to generate the desired outcomes, intention plays a crucial role.

Note that even though the distinction between intentional and stimulus-driven action is theoretically useful, human actions are rarely ever completely internally or externally guided, but are rather on a continuum between the two extremes (Krieghoff et al., 2011). Moreover, intentional action should not be treated as a unitary concept, but should be considered in terms of decisions regarding which action to execute (*what* component), at which moment in time (*when* component), and whether to execute an action at all (*whether* component; Brass & Haggard, 2008). Even though research on the ideomotor theory mostly investigates the *what* component, each of the three decision types have been associated with specific patterns of hemodynamic and electrophysiological brain activation (for a comprehensive review, see Brass & Haggard, 2008). For instance, Krieghoff et al. (2009) showed that the rostral cingulate zone (RCZ) seems to be related to the *what* component, while the *when* component was associated with activation in a part of the superior medial frontal gyrus. Further corroboration based on a more recent meta-analysis suggests that the different components of intentional action are indeed associated with specific neural underpinnings that are partially independent (Zapparoli et al., 2017).

To conclude, the ideomotor model drove considerable research on intentional action and action-effect anticipation in the past two decades. Previous reviews cover general behavioural, functional, and neuroanatomical mechanisms (Krieghoff et al., 2011; Nattkemper et al., 2010; Waszak et al., 2012). In the following sections, we discuss the role of intentional action in the specific context of auditory processing. We mostly focus on EEG studies reporting action-related prediction effects on early and mid-latency auditory event-related potentials (ERPs; i.e., N1, P2, MMM, N2, and P3 components), but we also include a few related behavioural, transcranial-magnetic stimulation (TMS), MEG, and fMRI studies.

## Auditory attenuation and the forward models

In 1973, Schafer and Marcus showed for the first time that tones generated by the participants themselves were followed by attenuated brain responses, compared with tones that were generated externally. This difference was obvious in the ERPs, in between the N1–P3 latency range (i.e., in between about 90 and 400-ms poststimulus presentation), and was largest over the vertex electrodes (Schafer & Marcus, 1973). Since its discovery, this commonly named *self-generation effect* appears to be a robust phenomenon at a neural level (i.e., as indicated by a considerable amount of neurophysiological studies), while at a phenomenological level, the comparably fewer studies point to somewhat mixed findings.

**Fig. 1:**
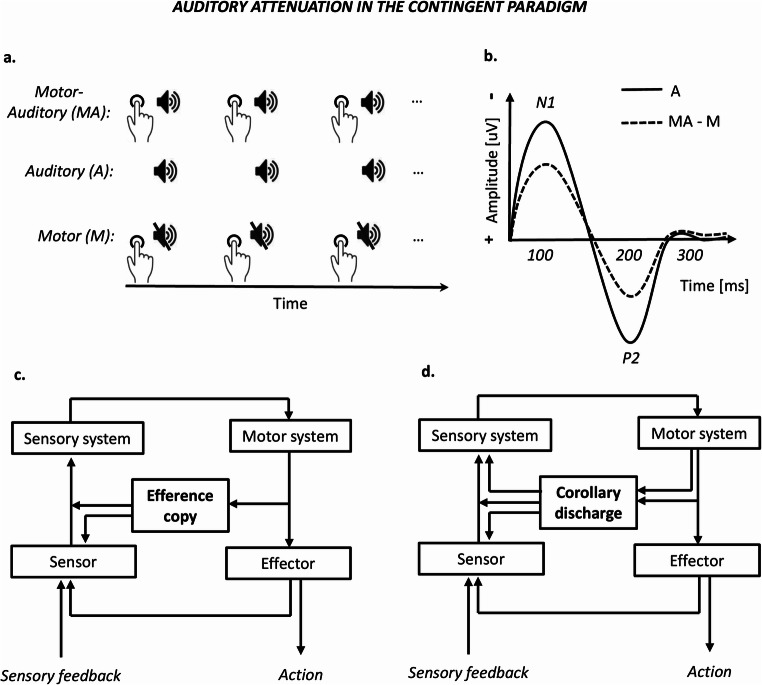
Paradigm and proposed mechanism behind the auditory attenuation. This phenomenon is typically studied in the contingent paradigm (**a**) where the comparison of the motor-corrected self-generated (MA–M) and externally generated (A) tones allows observing an amplitude attenuation following the self-generated sounds, typically for the N1 and P2 ERP components (**b**). The attenuation is taken to reflect the working of an internal forward model that determines whether the predicted sensory feedback, estimated based on an efference copy of the action’s motor command (**c**), and the received sensory feedback correspond to each other. Besides the efference copy, the corollary discharge represents a more general motor-to-sensory signal that operates within the forward model (**d**). (Figures 1c–d have been adapted based on Crapse & Sommer, 2008)

At a neurophysiological level, the observed attenuation effect following self-generated tones (e.g., Aliu et al., 2009; Martikainen et al., 2005; Timm et al., 2013; for a review, see Horváth, 2015; Hughes et al., 2013a) is typically investigated using the contingent paradigm (see Fig. 1a), which consists of three main experimental conditions (Horváth, 2015): motor-auditory, auditory, and motor. The attenuation effect is observed when comparing the motor-auditory (self-generated) and the auditory (externally generated) conditions, while the motor condition is necessary in order to subtract the motor-evoked potentials from the motor-auditory condition and thus make it comparable to the auditory condition. In the motor-auditory condition, participants are asked to perform an action, which is shortly followed by a tone. In the auditory condition, the same tone is passively presented at the same intersound interval (ISI) as in the motor-auditory condition. Finally, in the motor condition, participants perform the same action as in the motor-auditory condition but no tone is presented. The preferred action is typically a button press; however, other studies have also used foot-presses (Van Elk et al., 2014) and eye saccades (Mifsud et al., 2016). The attenuation effect appears earlier and larger when the tone is presented immediately after the action, while increased temporal delays result in later and/or less attenuation (Oestreich et al., 2016; Pinheiro et al., 2019; Van Elk et al., 2014). However, this positive relationship between action-effect proximity and attenuation magnitude does not seems to characterize high-schizotypy subclinical participants (Oestreich et al., 2016), while in patients with schizophrenia, short temporal delays might even increase the attenuation effect (Whitford et al., 2011). Thus, the magnitude of the neurophysiological attenuation as a function of action-effect timing might represent a good measure of psychosis.

Regarding the order of the experimental conditions, they are usually administered in separate blocks, where the motor-auditory condition is presented before the auditory condition (to allow comparable ISIs). Yet, in one study, the motor-auditory (self-generated) and auditory (externally generated) conditions were combined within the same block. In this mixed condition, the reported attenuation was even larger than when the self-generated and externally generated tones were administered separately, the authors concluding that this difference reflects the system’s ability to further distinguish between the sensory consequences of one’s own actions and those of others (Baess et al., 2011).

The contingent paradigm allows observing suppressed responses in the self-generation compared with the external-generation condition, typically at the level of the auditory N1 component referring to the first early negative response peaking around 100 ms after stimulus onset, and the P2 component representing a positive peak around 200 ms after stimulus onset, both having a frontocentral topography (e.g., Mifsud et al., 2016; SanMiguel, Todd, et al., 2013; Timm et al., 2014). The suppression of these components (see Fig. 1b) is typically taken to reflect internal forward modelling. More specifically, when an action is performed, an efference copy of the respective motor command containing information about the expected sensory feedback is presumably sent to the sensory systems (see Fig. 1c). This copy is compared with the actual sensory feedback, leading to attenuated sensory responses if they match (Blakemore et al., 2000; Crapse & Sommer, 2008; Miall & Wolpert, 1996). The term *efference copy* is related to the concept of *corollary discharge* representing a more general motor-to-sensory signal (see Fig. 1d) that may arise at various levels of the motor-sensory pathway (Crapse & Sommer, 2008). Recent data from Li et al. (2020) suggested that the two differ in specificity, with corollary discharge signalling preparation for a motor task without a specified content, while the efference copy signals preparation to produce specific effects, such as a spoken syllable. Nevertheless, the implicit definition of forward modelling typically includes both signal types, which often remain undissociated.

We would like to point out here that ideomotor theory, described above, and computational models using forward modelling—although very similar in spirit in that they emphasize the close relationship of perception and action—are arguably not based on identical concepts. Ideomotor theory assumes that actions are selected by means of the anticipation of desired outcomes, with the internal representation of the desired outcome, or goal, and the internal representation of the action that achieves this goal being one and the same. Ideomotor theory, thus, does not need to postulate that the action outcome is predicted, as the anticipation of the action outcome is already part of the selection of the action, or rather, the anticipation of the action outcome is the selection of the action (see, for example, Janczyk & Kunde, 2020; Waszak et al., 2012). Accordingly, in some accounts, sensory attenuation has been attributed to this preactivation of action outcomes in the sense of ideomotor theory, even though this has been mostly discussed in relation to visual predictions (e.g., Roussel et al., 2013; for a review, see Waszak et al., 2012).

The distinction between forward modelling and action outcome anticipation notwithstanding, N1 and P2 attenuation are often discussed together and it has been suggested that they might reflect different processes (Crowley & Colrain, 2004). This distinction is for instance supported by studies on patients with cerebellar lesions, for which the N1, but not the P2 attenuation to self-generated tones seems to be impaired (Knolle et al., 2012; Knolle et al., 2013). As the cerebellum likely represents the site of the forward model (Shadmehr et al., 2010), lesions at this level cause disruption of the forward model, which is in turn reflected in the lack of N1 attenuation. Additional results indicate that the N1, but not the P2 self-generation effect is likely to be superimposed by processes related to attention (Saupe et al., 2013). These findings thus suggest that while the N1 might be related to automatic processing, the P2 might reflect higher cognitive processes such as the processing of complex tone features (Shahin et al., 2005) and conscious evaluation of action outcomes. Altogether, the P2 might represent “a more direct measure of sensory-specific predictions,” as proposed by SanMiguel and colleagues, who reported that the P2 (unlike the N1) attenuation effect did not decrease with shorter stimulus onset asynchronies (SanMiguel, Todd, et al., 2013).

In addition to the N1-P2 complex consistently discussed in self-generation studies, one study also reported attenuation at much earlier stages, in the middle latency response (MLR), at the level of the Pa (i.e., a positive peak at about 27–33 ms post tone onset), and the Nb (i.e., a negative peak at about 40–46 ms post tone onset) components (Baess et al., 2009). While it is unclear if, or to what extent, the MLR effects can be taken to reflect predictive processing in the same way as the N1–P2 components, it appears that the mechanism driving sensory attenuation is robust and modulates a number of steps in the auditory processing hierarchy. Finally, note that the contingent paradigm has been mostly implemented to study poststimulus effects. Yet, a few studies looking at the readiness potential (RP), a negative ERP component which indexes action preparation, found that the RP was increased in the motor-auditory by comparison to the motor condition, thus indicating that the expectations regarding the self-generated tones are already represented at pre-stimulus levels (Pinheiro et al., 2020; Reznik et al., 2018).

As pointed out above, a disruption of this motor prediction mechanism due to cerebellar lesions leads to reduced attenuation (Knolle et al., 2012; Knolle et al., 2013). This seems to be the case for several other clinical populations such as schizophrenia (Ford et al., 2014) or autism spectrum disorder (van Laarhoven et al., 2019), in which impaired motor-to-auditory communication likely accounts for this deficit. Thus, the connection between our motor intentions and sensory processing is not just theoretically interesting, but also highly relevant from a clinical perspective.

Lastly, at a phenomenological level, a few studies that have looked at perceptual loudness judgements in the context of the self- vs. external-generation comparison, lead to somewhat conflicting results. On the one hand, a few studies have found that participants perceived the loudness of a tone attenuated when they generated the tone themselves, but not when the tone was computer-generated or generated by another person, complementary to the ERP results described above (Weiss et al., 2011; Weiss & Schütz-Bosbach, 2012). On the other hand, additional results indicated that perceptual attenuation also occurred for tones generated by another person (Sato, 2008), and even that the self-generated sounds were better detected (by contrast to attenuated; Reznik et al., 2014). Reconciling findings from Reznik et al. (2015) demonstrated that when the intensity of self-generated sounds was low, the perceived loudness was enhanced, and vice versa, high intensity of self-generated sounds was associated with an attenuated perceived loudness. Thus, unlike the consistent findings indicating attenuation in electrophysiological studies, the perceptual attenuation results are less conclusive. Studies simultaneously investigating neural and perceptual attenuation are rare. One recent set of results from Reznik et al. (2021) indicated an inverse relationship between the perceptual and neural responses: by comparison to externally generated sounds, self-generated sounds were associated with an increased detection sensitivity (i.e., enhancement) and decreased neural responses (i.e., attenuation). However, more research is needed to understand this apparent neural–perceptual discrepancy, particularly because the effect sizes reported by Reznik et al. (2021) are small.

### Is the attenuation effect specific to intentional action?

The sensory attenuation studies described so far demonstrate that the auditory consequences associated with our own actions are processed differently, compared with externally generated consequences. However, they do not demonstrate, first, whether these effects arise because of motor acts in general or because of intentional actions in particular, and second, whether the specificity of the action effects is important or the attenuation would occur to unpredictable action–effect associations too. The role of intentionality can be tested by contrasting brain responses to tones produced by voluntary vs. involuntary actions. Following this reasoning, Timm et al. (2014) recorded ERPs to sounds that were generated either by participants’ free button presses, or by finger movements initiated by stimulating the corresponding region of the motor cortex via transcranial magnetic stimulation (TMS). They found that the typical N1-P2 attenuation pattern only occurred following voluntary movement execution, and not in the stimulation condition in which no motor plan was available. Similarly, recent data from Jack et al. (2021) indicated that sounds following button presses caused by applying either electrical stimulation of the median nerve or manual force to the participants’ finger, did not lead to N1 suppression, by comparison to sounds following voluntary actions. Together, these results indicate that movement intention is a crucial element for the effect to occur (for additional TMS findings pointing to the role of intentional action for attenuation in the somatosensory system, see Haggard & Whitford, 2004; Voss et al., 2006). In line with this, results from Desantis et al. (2012) showed that participants perceived tones to be attenuated if they believed that the tones were self-generated, thus indicating that not just the motor act, but also the perceived authorship plays a role. Regarding the specificity of action–effect predictions, Baess et al. (2008) examined the magnitude of the N1 attenuation when two features, the frequency and the onset of self-generated tones, were either predictable or unpredictable. Specifically, these two features could be both predictable, both unpredictable, or one predictable while the other was not, yielding the corresponding four experimental conditions. Interestingly, these authors observed significant attenuation effects in all four conditions, while the largest suppression effect indeed occurred when both frequency and onset were predictable. This indicates that the prediction mechanism is more efficient if both the specific identity (*what* component of intentional action, see previous section), as well as the precise timing (*when* component) of the tone can be determined. However, the fact that attenuation was observed in all four conditions suggests that predicting one component only is sufficient to yield attenuation. The existence of *what-* and *when-*related processing has been also demonstrated in relation to the temporal precision and content specificity of inner speech (Jack et al., 2019), as well as in relation to the auditory sensory processing outside the context of action (Hsu et al., 2013).

Considering these results, one could conclude that intentional movement execution is necessary for the attenuation effect to occur, both if *what* and *when*, or only if *what* or *when* can be predicted. However, these conclusions are challenged by results coming from studies using the coincidence paradigm (Horváth, 2013; Horváth et al., 2012). In this paradigm, tones and key presses are only contiguous (i.e., the tone is not dependent on the key press, but the two co-occur, “by coincidence”). In this setting, Horváth et al. (2012) showed in a series of three experiments that the N1 and P2 components (as well as their counterparts in the MEG) were attenuated when the sounds coincided with the button presses, showing that action-effect causality was not a necessary condition for auditory attenuation to occur, but that the mere temporal coincidence between an action and a sound were sufficient to generate auditory sensory attenuation. This conclusion is of course incompatible with the idea that internal forward models and intention are causing the attenuation.

Taken together, the mixed evidence from these studies does not offer a coherent picture of the precise roles that intentional action and action-effect prediction play in sensory attenuation. Moreover, the attenuation effect is not specific to self-produced action effects. Recent studies have shown evidence of N1 attenuation both when participants initiated actions to produce sounds themselves and when they observed on a screen action effects that were produced by somebody else (Ghio et al., 2018; Poonian et al., 2015). Follow-up results nevertheless indicated that the N1 (but not the P2) attenuation might still be specific to own actions, when the timing of the self-performed and observed actions were better matched through a “live” observation context (by contrast to presented on a screen; Ghio et al., 2021). Furthermore, the attenuation effect is not even specific to action-related effects so that, for instance, N1 attenuation has also been reported for tones whose identity could be predicted based on the preceding sensory regularity pattern (Lange, 2009), or when a tone onset could be predicted on the basis of preceding visual information (Vroomen & Stekelenburg, 2010). In this regard, a recent self-generation study further showed that when the externally generated tones were cued by a countdown, the attenuation effect was reversed (i.e., was larger for the externally generated, than for the self-generated tones; Kaiser & Schütz-Bosbach, 2018; however, for contrasting results, see Klaffehn et al., 2019). To conclude, while the sensory attenuation studies have extensively shown that sounds associated with our intentional actions are processed differently than those that are not, the attenuation effect is not specific to intentional action, but seems to reflect a broader (unspecific) prediction mechanism.

### Sensory attenuation beyond the self vs. other comparison

An approach of studying action-related auditory predictions that is complementary to the experiments described in previous sections consists of comparing the auditory consequences of actions that are congruent with the expected action effects, with those that are incongruent. This approach has its origin in the fact that, when studying sensory attenuation, be it in relation to intentional action or to other phenomena such as visual-auditory (Vroomen & Stekelenburg, 2010) or auditory regularity-based predictions (Lange, 2009), a number of potential confounding factors need to be taken into account: temporal prediction and temporal control (the ability to predict and control the exact point in time at which a stimulus will occur), and motor and non-motor identity predictions (the ability to predict exactly which stimulus will occur, regardless of whether this is determined by the agent’s actions or not; Hughes et al., 2013a).

Following this reasoning, Hughes et al. (2013b) designed a paradigm where participants produced hand-specific tones by inversely associating the left and right hands with high vs. low pitch tones. In a predictable condition, each middle-finger key press generated a specific tone with 100% probability (low or high, according to the designed associations). In an unpredictable condition, the index-fingers key presses generated unspecific outcomes, that is, high and low tones with equal probability. Thus, the tones could be predictable vs. unpredictable (i.e., generated by the middle vs. index fingers), or prediction-congruent vs. prediction-incongruent (i.e., averaged across both fingers, a tone was presented frequently, and the other one rarely). When comparing the N1 ERP responses following the predictable vs. unpredictable tones, the authors did not observe any differences, by contrast to the sensory attenuation studies reporting differences between the self-generated (i.e., predictable) and externally generated (i.e., unpredictable) tones. Interestingly, the prediction-congruent by contrast to the prediction-incongruent tones (i.e., the frequent vs. rare action effects for each hand) elicited an attenuated N1. This result could also be interpreted as enhanced N1 for the incongruent tone in the sense of action-effect violation (in the same vein with the results from the active mismatch paradigm that we discuss in the next section). More recently, Heins et al. (2020) compared in an fMRI study two situations that resemble more closely what intended vs. unintended action effects could look like in real-life situations. They trained participants to perform two rather complex actions in the lab: tap dancing, in which case the action sounds were purposefully produced, and hurdling, where the action sounds were rather incidental, or a by-product of action. Videos of each participant performing both actions were recorded, while in a subsequent fMRI scanning session, participants watched the videos referring to their own actions, including action sounds. Importantly, the results showed that the incidental hurdling, by comparison to the intentional tap-dancing sounds, led to larger auditory cortex activation, in accordance with the sensory attenuation hypothesis postulating that intended action effects lead to suppressed auditory responses. Therefore, based on Hughes et al. (2013b) and Heins et al. (2020), we could conclude that intentional action and identity-specific action–effect predictions do generate auditory attenuation (see also Dogge, Custers, et al., 2019), even when controlled for differences related to temporal predictions and temporal control.

Therefore, integrating all evidence, it seems like attenuation can occur because of intentional action, but is not specific to it, as it might in fact not even be specific to action at all. Instead, it likely reflects a prediction mechanism that includes, but is not limited to forward modelling. Thus, in order to have a better understanding of how a person’s action intentions modulate the processing of incoming stimulation, we will have to look beyond the forward models, or, at best, consider forward models as part of more comprehensive theoretical frameworks. Therefore, predictive coding and/or the ideomotor theory might offer a clearer perspective on action intention-related predictions (see also Dogge, Custers, et al., 2019).

## The active oddball paradigm: Match vs. mismatch of expected action effects

Oddball paradigms are frequently used to study predictive processing in the auditory system. Two components are of particular interest in this context. First, the MMN that represents the difference between regularity-conforming standard tones and regularity-violating deviant tones. MMN is typically observed between 100 and 250 ms after stimulus onset and is considered to index the updating of the predictive model (for reviews, see Garrido et al., 2009; Näätänen et al., 2007). Second, the P3 component that is larger in response to deviant stimuli and is typically observed between 250 and 600 ms after stimulus onset. Being subsequent to MMN, P3 is thought to represent the next step in the auditory processing hierarchy (Horváth et al., 2008) and to index the allocation of involuntary attention to significant stimulus events (Nieuwenhuis et al., 2011). Furthermore, two P3 subcomponents have been distinguished, namely the fronto-central P3a and parietal P3b, presumably reflecting stimulus-driven orienting of attention to novel stimuli, and task-related orienting of attention that subsequently aids memory processing for task-relevant stimuli, respectively (Polich, 2007). We next discuss a series of studies that implement active variants (i.e., active generation) of the oddball paradigm to investigate sensory processing associated with intended (expected) vs. unintended (unexpected) action effects.

### Unexpected action effects trigger attention

Early results by Nittono and colleagues (Nittono, 2004, 2006; Nittono et al., 2003; Nittono & Ullsperger, 2000) showed that deviance detection is enhanced in active oddball tasks. In one study, the authors measured ERP responses to standard 1000-Hz tones, intermixed with 2000-Hz target tones (for which responses were required), and novel deviants of varying frequencies (Nittono & Ullsperger, 2000). In three different conditions, the tone sequence was either generated by one voluntary key press performed about every other second, presented passively using the same intervals as in the voluntary condition, or presented passively using a fixed interstimulus timing of 2 seconds. The amplitudes of the P3 component following both novel deviants as well as target tones were larger in the voluntary condition, as compared with both passive conditions (Nittono & Ullsperger, 2000). This pattern of results with overall larger P3 amplitudes following voluntary actions was replicated in the auditory as well as visual modality (Nittono, 2004; Nittono et al., 2003). However, in those studies (Nittono, 2004; Nittono & Ullsperger, 2000) the perceptual differences between tones were sufficiently large so that both the deviant, as well as the target tones, appeared to be highly salient relative to the standard tones, thus making it unclear whether intentional actions benefit saliency detection, or other task-relevant processes. To this end, Nittono (2006) implemented a task similar to the one previously described, with the only difference being that standard and target tones were perceptually close (1940 Hz and 2000 Hz, respectively), and that the deviant tone was easily distinguishable from both the standard and the target tones (500 Hz). In this context, the ERP results showed that the deviant-related P3a (high saliency) but not the target-related P3b (low saliency) was larger in the voluntary than in the passive condition. In line with recent views according to which stimulus saliency modulates perception and memory (Mather et al., 2016), these results indicate that the extent to which intentional actions improve the detection of unexpected sensory consequences depends on the stimulus’ physical properties.

However, note that this series of studies comes with a serious limitation: like the attenuation studies discussed above, the comparison of motor vs. non-motor predictions likely confounds a number of processes (Hughes et al., 2013a). In this regard, the direct comparison (without prior subtraction of the motor-related potentials) between the ERP components in the active vs. passive conditions is particularly problematic. Moreover, the P3 component, which is central in the results described above, has been consistently described in relation to attention allocation (Polich, 2007), which is likely to differ between active and passive tasks. Nevertheless, despite these limitations, these results provided an important starting point for further studies aiming to investigate the effects of intentional action while controlling for these potentially confounding factors.

One such example comes from Waszak & Herwig (2007) who, in fact, used the same task and stimuli as in Nittono (2006), with one crucial difference: in the acquisition phase, participants learned that a left key press triggered the lower frequency tone (500 Hz), and a right key press triggered the higher frequency tone (1940 Hz). In the subsequent test phase, participants were required to press the two keys equally often; regardless of whether they pressed the left or the right key, any of the 1940-Hz standard, 500-Hz deviant, or 2000-Hz target tones could be presented (with a probability of 75% for the standard, and 12.5% for the deviant and target, respectively). Results showed that the P3a response to deviant stimuli was larger when triggered by the key press that in the acquisition phase resulted in the standard tone, compared with when triggered by the key press that in the acquisition phase resulted in the deviant tone. Importantly, in line with the ideomotor principle, the authors interpreted the differential processing of deviants that either matched or mismatched the previously learned action–tone associations to reflect an effect of intentional action, where the violation of intended action effects draws attentional resources towards the unexpected effect (Waszak  & Herwig, 2007). Thus, the initial conclusion of Nittono and Ullsperger (2000) according to which learned action effects improve deviance detection seems to stand true, also when controlling for the potentially confounding processes described above.

### Preattentional processing levels: The intention-based MMN

Thus far, we have seen that in active variants of the oddball paradigm the top-down effects of action intention occur at relatively late auditory processing levels associated with attention orientation towards the action outcomes. What about preattentional, earlier processing levels? Although it has been argued that top-down action–effect predictions “do not affect the MMN-generating process” (Waszak & Herwig, 2007; for similar arguments, see Rinne et al., 2001), one shortcoming is that the impact of alternative sources of prediction (such as those extracted from stimulus regularity) on the action-effect expectations, have thus far not been considered with the active variants of the mismatch paradigm. In order to get a more accurate picture of the action–effect predictions on the early auditory processing, the potential impact of tone regularity should thus be additionally examined.

**Fig. 2:**
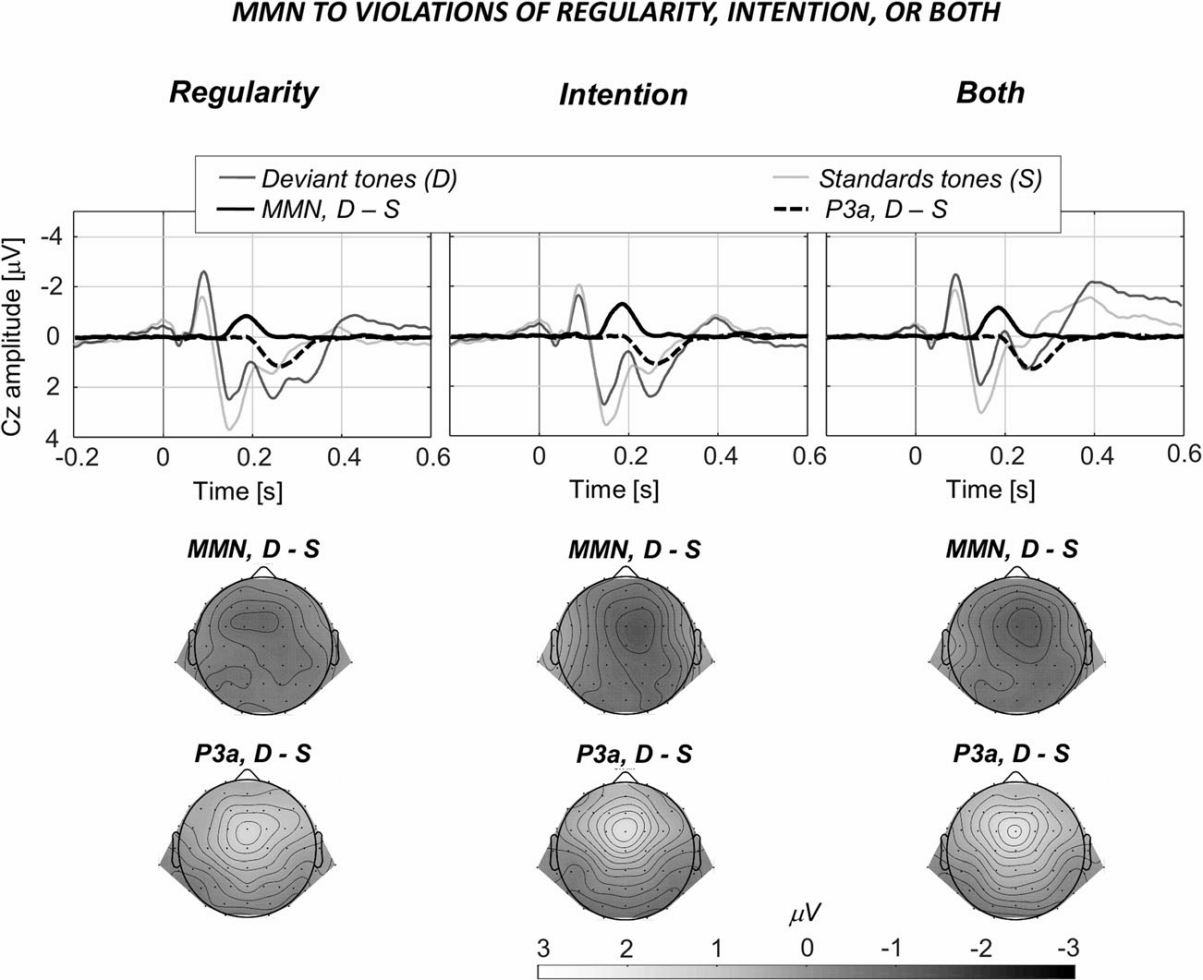
MMN and P3a results of Korka et al. (2019). By manipulating the associations between left and right key presses and high and low pitch tones, Korka et al. (2019) compared predictions based on tone regularity, intention to generate a specific tone, or both combined. Similar MMN and P3a responses have been observed in all three conditions. The dark lines represent the difference activation (deviant – standard) for the components representing the MMN (full lines) and P3a (dashed lines) responses, as identified by a temporal principal component analysis, which was used to separate the ERP wave into components of interest. The coloured/grey lines represent the sum of all retained principal components corresponding to the grand-average ERPs, for the standard and deviant tones. Importantly, in the Intention condition, there was no global regularity pattern, meaning that the MMN and P3a were elicited purely due to the violation of expected action effects. (Figure adapted based on Korka et al., 2019)

With this goal in mind, Korka et al. (2019) asked participants to make left and right key presses to generate high and low frequency tones. Based on the key–tone associations, but also on the ratio of left and right key press, they contrasted in three conditions predictions based on tone regularity, action intention, or both combined. Specifically, in the intention and regularity conditions, the keys were pressed with equal chances to generate either hand-specific tones of high and low frequency, or the same regular tone (presented regardless of the action choice). In a third condition combining both intention and regularity, one key was pressed more frequently than the other one to generate tones, which were hand specific, but also regular. In all conditions, violations occasionally occurred by presenting the other tone—that is, if a low tone was to be expected based on either intention, regularity, or both, a high tone was rarely presented instead. The results showed that the sensory-specific N1b and Tb components of the N1 response were modulated by regularity only, suggesting they reflect neural adaptation; however, the MMN and P3a components—which are also displayed here in Fig. 2—were similarly elicited in all three conditions referring to violations of regularity, of intention, or violations of both regularity and intention combined. Korka et al. (2019) thus concluded that in the context of action–effect predictions, the contributions of intention and regularity can be partly dissociated in the sense that they both produce mismatch effects; however, when both prediction sources are available, they presumably integrate, rather than add up.

Importantly, the MMN and P3a effects (see Fig. 2) observed by Korka et al. (2019) in the intention condition were elicited without a global regularity context (i.e., when the two tones, besides being predictable or unpredictable based on the action–effect associations, were overall presented with equal chances). Thus, the effect of intention-based top-down predictions was evident at early and later processing levels, without the potentially confounding effects of neural adaptation due to regular input. Note that the interpretation that the N1 component reflects low-level sensory adaptation, while the MMN and P3a components index predictive processing in the auditory system, is congruent with recent results from Quiroga-Martinez et al. (2020), who showed that the magnetic counterparts of these components are modulated by either sensory adaptation (N1m) or probabilistic predictions (MMNm, P3m). However, whether the N1 component is modulated by stimulus-specific adaptation or by other prediction-related processes seems to vary across paradigms (see, for instance, Hughes et al., 2013b, and the “sensory attenuation beyond the self vs. other comparison” section in the present work). SanMiguel, Todd, et al. (2013) argued that in sensory attenuation studies, the unspecific rather than the modality-specific N1 attenuation is typically observed. Thus, stimulus-specific adaptation or other processes might be differently reflected in the N1-subcomponents at play.

The existence of intention-based effects at the MMN levels is further congruent with recent results. First, Korka et al. (2021) showed that a higher-magnitude MMN was obtained if two standard tones enclosing the pitch of a rare deviant were associated with the left and right key presses, by contrast to when both key-presses generated both standard tones equally likely (i.e., in the absence of any specific action–effect associations). Thus, the MMN component reflects that action–effect predictions improve the encoding of the stochastic sequences (referring to basic probabilities of the standards vs. deviant), a type of regularity that is otherwise difficult to encode at early processing levels (Schröger & Roeber, 2021). The results of Korka et al. (2021) also suggest that the N2, a further mismatch-related component presumably reflecting higher-order prediction violation (Folstein & Van Petten, 2008), was only elicited following the specific action–effect associations. Second, Le Bars et al. (2019) reported that a mismatch effect at the level of the N2b component was only generated when participants could freely choose their actions, but not when the action choice was externally cued. The distinction between the MMN and N2, or the N2b is that the later components might be more sensitive to conscious deviance processing, with the N2b being especially related to task-relevance and the monitoring of tones requiring further responses (such as is the case with catch trials implemented by Le Bars et al., 2019). Yet, all these components belong to the same family of processes related to deviance processing and error detection (Folstein & Van Petten, 2008). Therefore, taken together, this set of recent results demonstrate that mismatch detection, a phenomenon that was until recently exclusively related to environmental regularities, can be additionally modulated by intentional action and action–effect predictions. Besides mismatch detection, the results by Korka et al. (2021) indicated that specific action–effect predictions also modulate the activity observed at prestimulus levels: a larger and earlier lateralized RP (LRP) was elicited if left and right key presses were each associated with a specific tone, by contrast to when both key presses equally generated both tones.

Earlier results from Band et al. (2009) further support the notion that violating action–effect predictions leads to mismatching-like effects. These authors implemented a probabilistic learning task where, similarly to Korka et al. (2019), low-probability and high-probability tones were inversely associated with the left and right hand. Importantly and unlike in Korka et al. (2019), these associations were irrelevant in relation to the subsequent visual categorization task that participants had to perform, thus leading to the possibility of evaluating both the explicit performance-related feedback from the main visual task, as well as the processing of presumably task-irrelevant action effects. The authors found an action-effect negativity component, with larger negative responses peaking around 200 ms following low compared with high probability action effects, very similar to the feedback-related negativity, a component associated with action monitoring and feedback processing, in both the auditory (e.g., Kim & Arbel, 2019) as well as the visual (e.g., Bellebaum & Colosio, 2014; Yeung et al., 2005) systems. This points to the fact that even irrelevant action effects lead to perceptual anticipations and that action–effect predictions do not only serve action selection, but also action monitoring, as in the case of explicit performance evaluation. While Band et al. (2009) do not discuss the observed auditory signals in terms of intention-based mismatch effect in these specific terms, due to the similarity of the implemented action–effect associations and observed signals relative to Korka et al. (2019), one could argue that both studies describe the same phenomenon.

In summary, the series of studies described in this section indicates that top-down predictions based on intention modulate several steps in the auditory processing hierarchy. Importantly, intentional action leads to reliable prediction effects also when controlling for tone regularity, while the relationship between intention and regularity as distinctive prediction sources varies from integration to possibly additivity, depending on the experimental context.

## Is there something missing here? Omission of expected action effects

**Fig. 3:**
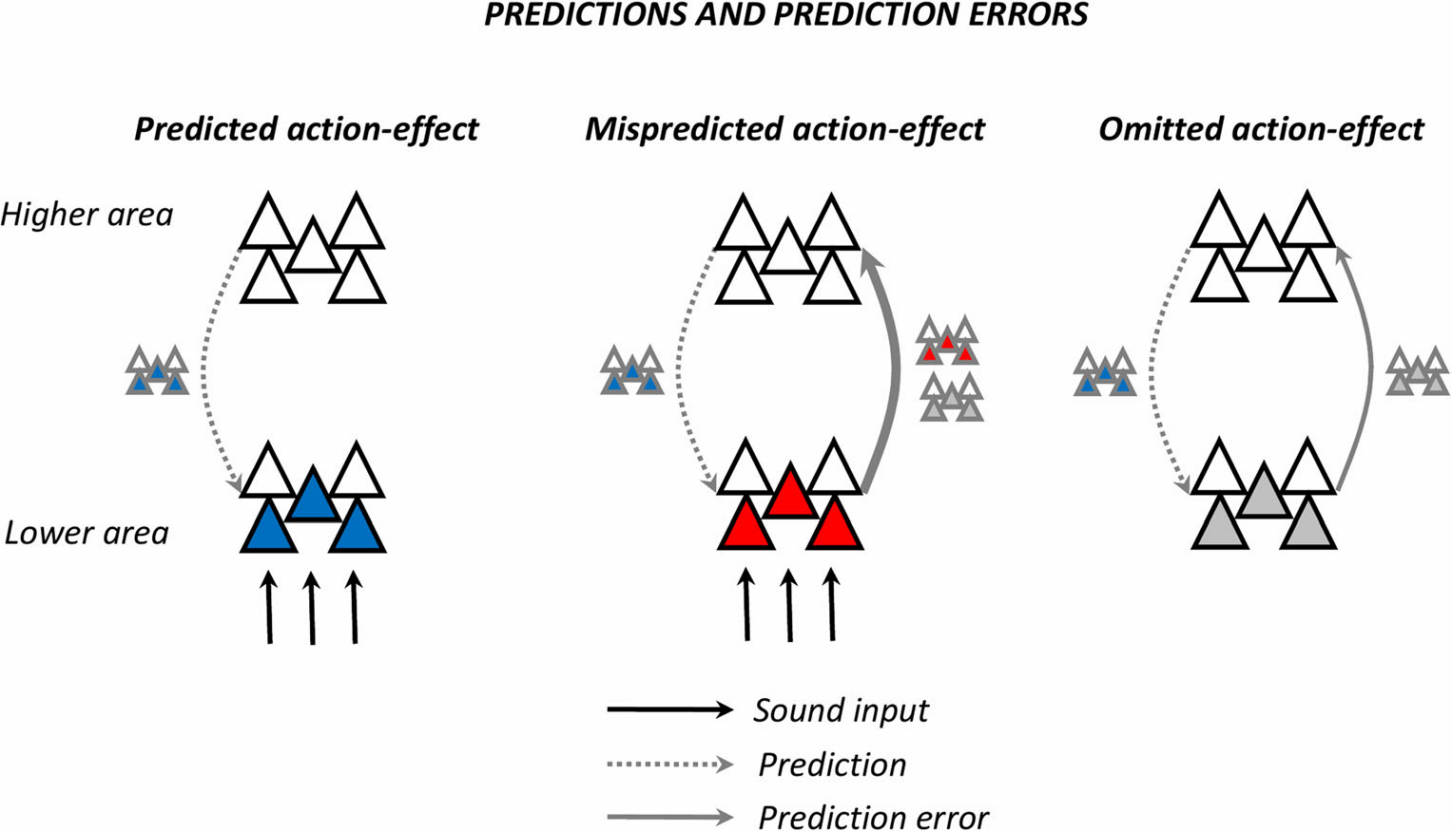
Schematic representation of predictions and prediction errors. The figure illustrates the transmission of information from higher to lower cortical sensory areas and vice-versa. When an action is performed, information regarding its predicted effect is sent from higher to lower cortical areas. Left: If the prediction is matched by the sound input, the prediction error is minimized. Middle: In case of mispredicted sounds, the prediction error likely overlaps signals related first, to input that has been delivered, but not expected, and second, to input that has been expected, but not delivered. Right: If the predicted input is omitted, this information is sent back up the cortical hierarchy as prediction error; thus, omission responses allow measuring the brain’s endogenous prediction-related signals. (Figure adapted based on SanMiguel, Widmann, et al., 2013)

In the previous sections, we discussed results from two main categories of studies: those describing the processing of tones following intentional actions by comparison to tones generated externally or by others, and those that compared expected vs. unexpected tones based on learned action effects. Omission designs, on the other hand, investigate brain responses following expected, but omitted stimuli, allow measuring the brain’s endogenous prediction-related signals (see Fig. 3); that is, prediction errors can presumably be directly measured due to preactivation in units that expect, but do not receive any input (Bendixen et al., 2009; Arnal & Giraud, 2012; SanMiguel, Widmann, et al., 2013). By contrast, in the case of mismatching (i.e., mispredicted) stimuli, the measured brain responses likely aggregate two different signal types: one related to input that has been delivered, but not expected (i.e., this is the case of the deviant tones), and one related to input that has been expected, but not delivered (Arnal & Giraud, 2012; Schröger et al., 2015). Omission designs thus have the potential of describing a more genuine picture when it comes to brain predictions, reason that drove considerable research from various domains and across sensory modalities (for a review, see corresponding section in Schröger et al., 2015). Here, we discuss a few electrophysiological studies along with a set of fMRI results underlying the effects of omitting the sounds expected based on intentional actions.

### Action-effect specificity determines the magnitude of the omission responses

SanMiguel and colleagues conducted two experiments showing that omissions of self-triggered tones elicited ERP responses similarly to those elicited by the sounds themselves (SanMiguel, Widmann, et al., 2013), the magnitude of these so-called omission responses further depending on whether the precise identity of the expected tones could be determined or not (SanMiguel, Saupe, et al., 2013) . Specifically, in the first study, participants generated the same click sound (or omissions) by pressing a key every 600–1,200 ms; in one condition, the sounds were presented in 88% of the trials, while in the remaining 12% of the trials, omissions occurred instead. In a second condition, the ratio of sounds-to-omissions was 50%–50%. Omission N1 (oN1) and omission N2 (oN2) responses only occurred following the rare omissions, where a stable association between the action (i.e., key press) and its associated effect (i.e., click tone) could be formed (SanMiguel, Widmann, et al., 2013). In the second study, participants again pressed keys to generate tones (or omissions); in one condition, the key presses produced a single sound (i.e., the same sound was repeated on every trial, as before), while in a second condition the key-presses produced random sounds, selected on every trial from a pool of 48 environmental tones. Omissions occurred on 13% of the trials in both conditions and triggered the oN1, oN2 and additionally, omission P3 (oP3) responses in the single sound condition, where both the tone’s specific identity as well as timing could be determined; this was, however, not the case in the random sounds condition, where only the tone’s presentation timing could be determined (SanMiguel, Saupe, et al., 2013). These two studies indicate that in the context of action, omission responses are only elicited if first, stable action-effect contingencies can be formed (i.e., the action generates the tone more often than it generates the omission), and second, the tone identity is specific and can be predicted on every trial (but see below for evidence suggesting that unspecific predictions are associated with omission responses too). Based on these results, SanMiguel, Saupe et al. (2013) concluded that the auditory system is organized serially, in a context where predictions based on the tone identity (predicting *what*) further determine the influence of temporal predictions (predicting *when*); in other words, if *what* cannot be determined, the effect of *when* is reduced.

Stekelenburg and Vroomen (2015) further aimed to evaluate the potential differences between motor-auditory vs. visual-auditory predictions on the processing of expected, but omitted tones. To this end, they implemented the same conditions as in SanMiguel, Widmann, et al. (2013), and additionally two visual conditions in which a clapping tone was associated with a video of a handclap; as before, the tones-to-omissions ratio could be either 88%-12%, or 50%-50%. The results showed no differences between the motor-auditory and visual-auditory conditions overall, suggesting that the two prediction types activate similar sensory templates, despite presumably having independent generators in the brain (i.e., motor vs. visual cortices, respectively). Further, in accordance with SanMiguel, Widmann, et al. (2013), the oN2 responses were larger following the rare omissions (in the 12% condition) for both prediction types, by contrast to the oN1 response, which, interestingly, was similarly elicited in both the 12% and 50% conditions, for the motor-auditory, as well as for the visual-auditory conditions. On the one hand, this different pattern of results for the oN1 and oN2 somewhat challenges the notion that stable (and specific) action-effect contingencies are necessary to form expectations, at least at very early sensory levels where chance associations might be sufficient to generate expectations. On the other hand, it supports the notion that the auditory prediction system is organized serially, meaning that components at different processing levels might be differentially modulated by the reliability and specificity of the action effects.

### The prediction system is flexible and tolerates unspecific action effects as well

Dercksen et al. (2020) recently aimed to replicate the results of SanMiguel, Saupe, et al. (2013) in an experiment that closely recreated the original one. The results replicated SanMiguel, Saupe, et al. (2013) regarding the single sound condition, as well as regarding the differences between the single vs. random sounds conditions. That is, oN1, oN2, and oP3 responses were reliably elicited in the single sound condition, and the magnitude of these components was larger than in the random sounds condition. However, Dercksen et al. (2020) additionally observed that the oN1 and oP3 components were reliably elicited following omissions of the random sounds as well, even if these were attenuated, compared with the omissions following the single sound. This was not the case for the oN2 that the authors in fact interpret as an omission mismatch negativity component (oMMN), which was reliably elicited in the single sound condition only, suggesting that at this processing step, a predictable sound identity is crucial. These results thus support and go beyond those obtained by SanMiguel, Saupe, et al. (2013) by showing that the predictions based on a specific tone identity play indeed a crucial role, but that a reliable tone identity is not mandatory to elicit prediction error. Instead, it seems that both specific and unspecific predictions (i.e., in which both *what* and *when*, or only *when* respectively, can be determined) are implemented in the auditory processing hierarchy, where precision weighting (i.e., the reliability/uncertainty attributed to the error units) likely determines the strength of the prediction error, which is in turn reflected in attenuated or enhanced omission responses.

Yet the aforementioned studies have one shortcoming: They compare two different contexts that are defined either by a single, repetitive sound, or by random sounds extracted on every trial from a 48-tones pool. It has been shown that such differences in stimulation influence the neural refractoriness states associated with different sound frequencies which, in turn, lead to differences in the N1 amplitude (Jacobsen & Schröger, 2001). Korka et al. (2020) addressed this potential confound by implementing an omission design with two conditions in which the tones’ identity was either predictable or unpredictable based on the specificity of the action–effect associations for left and right hands. That is, a left key press could generate Tone A and a right key press Tone B, or both key presses could generate both tones with equal probability. Importantly, these two conditions were physically identical in terms of stimulation and thus any differences in the omission responses could be exclusively attributed to top-down expectations based on the action choice and not to the differences in stimulation. An additional condition in which both the left and right keys generated the same tone was implemented, in order to test whether there are any processing costs/benefits associated with maintaining single vs. multiple tone representations. In agreement with previous studies, the results showed that indeed, the amplitude of the oN1 component was modulated by the specificity of the action-effect representations even when controlling for differences in physical stimulation between conditions. Moreover, the results showed that maintaining unique, compared with simultaneous representations, lead to an earlier oN1 response. Further, the oP3 component was similar regardless of the specificity or uniqueness of the action-effect representations. In sum, the results of Korka et al. (2020) point once again to a hierarchy of the action-related predictions in the auditory system. In this context, the specific identity of the expected action effects generates processing costs (for unspecific associations) or benefits (for specific associations) at the early auditory processing levels, while it seems that later processing levels reflect a more general expectation regarding the mere occurrence of a tone.

The action-related omission studies described thus far can be summarized as follows. Predictions based on intentional action have an effect on early and late processing levels in the auditory hierarchy. In this context, being able to determine the precise tone identity and timing of the expected input plays the most important role. However, the system appears to be highly flexible in tolerating imprecise expectations too. As pointed out by Dercksen et al. (2020), this idea is “more compatible with everyday life,” where imprecise predictions seem to be the rule, rather than the exception. The results in this section prove once again that intentional action is not a unitary concept, but one at the interplay of several components, among which *what* and *when* are particularly relevant (Brass & Haggard, 2008; Krieghoff et al., 2009).

### Expected action effects further determine auditory cortex activation to action alone

Kühn and Brass (2010) further tested the idea that action preactivates the expected sensory consequences using fMRI. Like in many studies before, they implemented an acquisition phase in which participants learned associations between actions and sounds and, additionally, between non-actions and sounds. That is, participants executed a two-choice reaction task, where they could either press a key, or decide not to press a key, while both the action and the non-action were associated with specific tones. In a test phase including fMRI scanning, participants could again choose whether to press a key or not, with the main difference that no effect tones were presented. In addition to these voluntary go/no-go decisions, two extra conditions were implemented, in which the go/no-go responses were cued by visual stimuli. The results indicated that, in the absence of any auditory stimulation, the auditory cortex was activated following the actions that previously produced tones, irrespective of whether these were self-chosen or cued. Supporting the idea that actions become associated with their consequences, these results demonstrate that the brain preactivates the template of the expected action effects, in agreement with the electrophysiological responses evoked by omitted, but highly expected action effects. Moreover, Kühn et al. (2010) obtained similar results for the visual modality as well, showing that the parahippocampal place area (PPA) and the fusiform face area (FFA) were activated following key presses that were previously associated with pictures of houses or faces, respectively.

Interestingly, the results of Kühn and Brass (2010) further showed that the auditory cortex was activated in response to the expected tones associated with self-chosen non-actions, while this was not the case for the cued non-actions. Importantly, this shows that what determined the preactivation of the expected sensory consequences was the decision to act or not to act, rather than the movement itself. In a sense, this is congruent with the previously discussed TMS results of Timm et al. (2014), who showed that the intention to perform an action, rather than the motor act itself (triggered by TMS pulse) was what caused the sensory attenuation, by comparison to tones generated externally (see “is the attenuation effect specific to intentional action?” section). To conclude, while we have previously discussed the roles of the *what* and *when* components of intentional action in relation to the auditory processing hierarchy, Kühn and Brass (2010) might have finally provided some direct evidence for the role of the *whether* component, namely that the bare decision of acting or not modulates the auditory responses according to the expected consequences of action or non-action.

## Actions are cued by learned action effects

We have seen in the previous sections of this review how several paradigms demonstrate that intentional action and learned action effects modulate the auditory processing of sounds when these are generated by the participants’ intentional actions. Let us now consider studies looking at brain responses when sounds that were previously associated with intentional actions are presented in the absence of the action itself. Musical sequences provide a useful framework for this purpose, as learning to play a musical instrument requires strong associations between very specific movements and musical notes (Herholz & Zattore, 2012).

In this context, Haueisen and Knösche (2001) used MEG to investigate patterns of motor activation in pianists vs. non-pianists while listening to well-known piano pieces. The results showed larger activation in effector-specific areas in pianists compared with non-pianists, presumably due to the action effects that pianists had previously acquired through extensive musical training. More recently, Stephan et al. (2018) trained non-musicians to learn simple melodies composed of sequences of four musical notes. Using TMS applied over the M1 area, the authors were able to show that the amplitudes of the motor-evoked-potentials (MEP) increased after the participants learned the melodies, by comparison to before. Importantly, this activation increase was observed before the tone onset and was effector-specific. These findings thus directly support the notion that once action–effect associations are built, the learned melodies cue the corresponding actions, even in the absence of movement.

Outside the context of music, a PET study by Elsner et al. (2002) showed that the caudal supplementary motor area and the right hippocampus increased their activity following sounds that were previously triggered by intentional actions, by contrast to neutral sounds without prior associations. Similarly, a TMS study by Ticini et al. (2012) indicated that the motor system precisely represents newly acquired action–effect associations, as the amplitudes of the motor-evoked potentials (MEP) were increased following the passive presentation of sounds which were congruent (by contrast to incongruent) with previously learned associations. Thus, these two sets of results further demonstrate that action effects that were previously associated with intentional actions cue motor activity in the brain, also in the case of more arbitrary action–effect associations. To conclude, in accordance with the ideomotor principle, learning action effects associations induces brain plasticity, reflected in motor-specific activation following sounds that have been previously associated with actions.

## Intentional action and auditory processing: Summary and proposed mechanisms

As described in the beginning of this review, the ideomotor principle has been used to formalize the concept of intentional action, the results from the auditory modality discussed above being largely in agreement with this. The model postulates that actions and their sensory consequences are encoded together; based on this, we select our actions in order to generate desired outcomes. Indeed, as we have seen with the active variants of the oddball paradigm, unexpected action sounds evoke larger responses at several steps along the auditory processing hierarchy than expected action sounds. The sensory attenuation studies showing reduced brain activity following intentional action and intended action effects are in further agreement with the ideomotor notion, even though results here are typically attributed to forward modelling (or neural preactivation). Note that the ideomotor principle and the forward models are not incompatible with each other: they both acknowledge the key role of action-effect anticipation, but according to the ideomotor principle, predictions rely on the person’s intentions, rather than on efference copies (Dogge, Custers, et al., 2019). Furthermore, several omission studies demonstrate the existence of a flexible system representing specific and unspecific action-effect expectations, in agreement with the notion that action intention is not a unitary concept, but can refer to predicting *what*, *when*, and *whether*. Finally, we have seen that the reverse is also true, that is, sounds alone cue the actions with which they have been encoded.

One shortcoming of the ideomotor principle is that it does not explain the precise mechanisms of adjusting action-effect expectations. That is, the existent representations are not permanent, but they constantly reorganize according to experience, context, and/or new evidence. In other words, the original ideomotor theory explains the feedforward connections generating predictions based on action intention, but it does not tell much about backward comparisons that allow the system to update. An advancement of the ideomotor framework, the updated theory of event coding (TEC V2.0) provides a more integrating view regarding the common codes of perception and action, according to which control processes such as persistence or flexibility of the associations (referring to strong vs. weak impact of the current goals) should additionally be considered (Hommel, 2019). From this perspective, the ideomotor principle becomes more functionally compatible with recent views explaining perception and action as inferential processes.

In this vein, we next present a model indicating how expectations at sensory levels are generated and updated based on intention and learned action effects. Extending on the auditory event representation system (AERS; Schröger et al., 2014; Winkler & Schröger, 2015), we propose the existence of a common predictive mechanism that receives information from the motor system that is in turn used to generate and adjust sensory perception, besides receiving sensorial information extracted from environmental regularities. Accordingly, the two information types (i.e., action-based and sensory-based) could be understood as two separate sources—one derived from an initial sound analysis and the other one derived from ideomotor learning—that feed into a common system. Importantly, this implies that the two information types can either integrate, have additive effects, or have independent effects on the final sensory representation, depending on the task circumstances.

### The auditory event representation system (AERS)

**Fig. 4:**
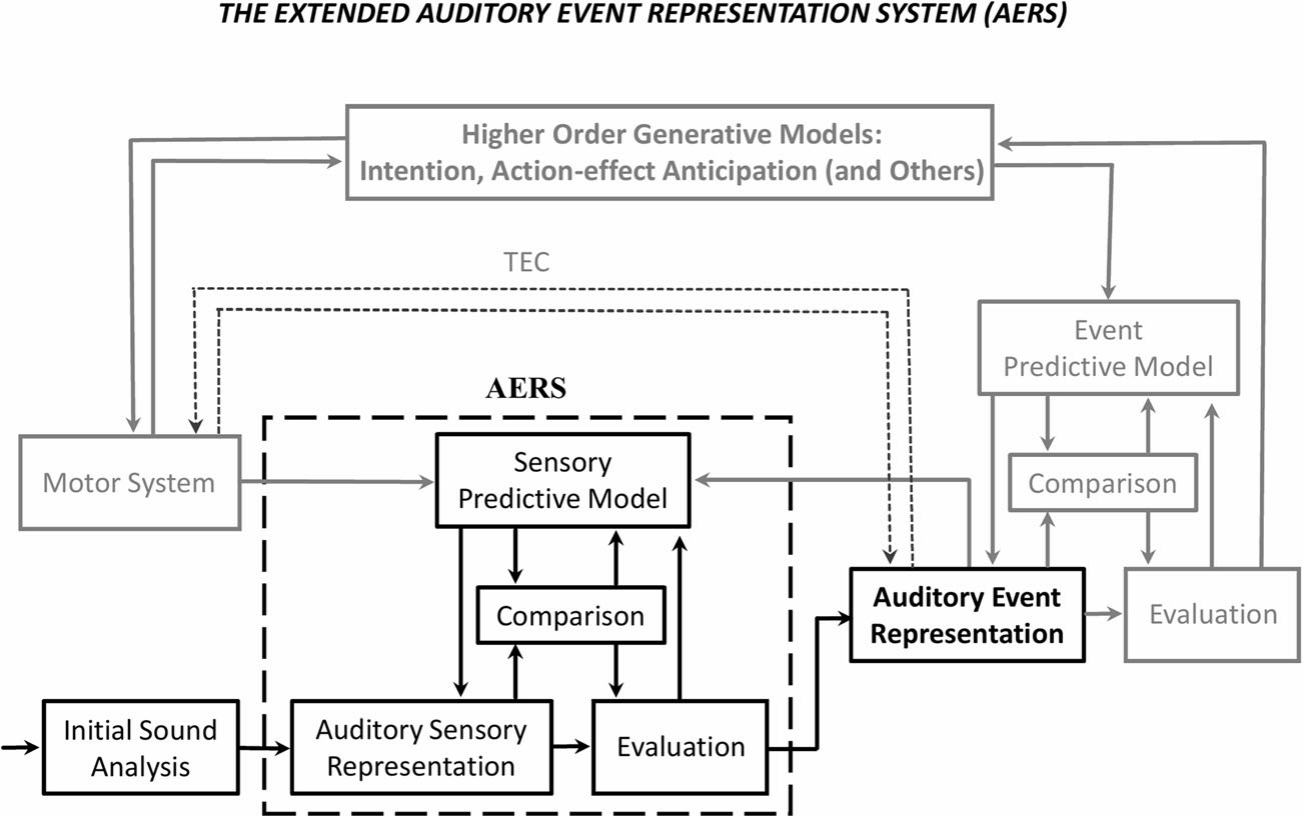
A model explaining the effects on action intention on auditory processing. First introduced to explain the auditory regularity-based predictions, we propose an extended version of the auditory event representation system (AERS; Schröger et al., 2014; Winkler & Schröger, 2015)*,* that explains the effects on action intention and action–effect predictions on the auditory processing. Represented in black is the original model as presented in Winkler and Schröger (2015), while the new elements referring to the motor input into AERS are represented in blue/grey. Dashed grey lines mark the model’s compatibility with the theory of event coding (TEC). (Figure adapted based on Winkler & Schröger, 2015)

First, we would like to describe the elements of the original AERS as proposed in Winkler & Schröger (2015), shown in Fig. 4, in black colour. The model assumes that information processing is directed towards the future. Representations of the known environmental regularities are created and maintained until new evidence becomes available. This is implemented by a system with module-like properties^1^ describing the manner in which the new auditory information is processed to create and update the predictive model. In this context, the basic auditory features of a given sound (such as frequency, loudness, timbre) represent the input. These are detected at an initial sound analysis step and are used to construct an auditory sensory representation that not only contain information regarding the basic auditory features of the incoming sound but also regarding the auditory context. For instance, in a simple oddball sequence, sensory memories lead to low-level expectations towards the presentation of a standard tone. These expectations are achieved with the help of the predictive model that stores information about the current applicable rule. Besides guiding the formation of the sensory representation, the predictive model also helps to compare the emerging representation with the current rule. This comparison might update the sensory representation, in case the incoming sound mismatches the predictive model (i.e., is a deviant). In this context, the MMN represents the marker of this updating step, which can also be understood as new information indexing the prediction error that is fed back up to the predictive model, where new rules might be extracted (i.e., model updating; Winkler et al., 1996). The outcome of the comparison along with the current version of the sensory representation further feed into the evaluation process that prepares the information for higher-level processes, such as attentional control indexed by the elicitation of the P3 component. The evaluation thus focuses on aspects of the sound that are outside the auditory environment, but might also initiate the search for new rules in the auditory environment, for example, in the case of successive deviant events. Finally, the AERS output consists of an auditory event representation, referring to both the sound-related information as well as the general context in which the sound was presented.

### Intention-based predictions and the extended AERS

In the context of AERS, we suggest that information from the motor system based on intention and ideomotor learning represents an additional type of input, which, if fed into the model, produces similar effects along the auditory sensory processing hierarchy as described above. These additions, creating the *extended AERS,* are represented in blue/grey colour in Fig. 4. More specifically, information referring to the intention-based expectations (i.e., acquired through the associations between actions and sounds via ideomotor learning) are stored into the motor system. The source of this information is represented by a higher order generative model storing information about intentions, action–effect couplings, or other types of information with predictive value. Note that this is in line with recent views, according to which the retrieval of information based on ideomotor learning is governed by “current metacontrol states” (Hommel, 2019). From this perspective, intention and ideomotor learning represent higher-level cognitive information, which, in agreement with the hierarchical nature of information processing in the brain (Friston & Kiebel, 2009), are transmitted to the lower sensory levels of AERS. Thereafter, once the motor system is engaged in producing an action sound, the corresponding information is passed through the sensory predictive model towards the initial auditory sensory representation, where an expectation towards the sound paired with that specific action first emerges.

An important aspect to be noted here is that the intention-based representations are formed in a top-down manner, while the previously described regularity-based representations arise because of bottom-up stimulation. That is, both information types eventually feed into the same sensory predictive model and contribute towards forming an initial auditory sensory representation, but the source is different (i.e., higher-level cognitive areas vs. peripheral, sensory memory areas). As before, a comparison between the emerging sensory representation and the current rule stored into the predictive model is performed at the sensory levels, a mismatch between the two potentially causing the model to update. As we have seen in previous sections, this can be indexed by mismatch responses (i.e., MMN, N2) or comparable omission-related components, elicited to violations of expected action effects. The outcome of the comparison along with the current version of the sensory representation are once again fed into the evaluation process, the studies reviewed in previous sections indicating that at this processing step, the P3 and omission P3 components are often elicited following unexpected action outcomes.

Finally, the auditory event representation stores the assembled information regarding the sound in relation to its associated context (i.e., action), which in turn feeds into a further comparison process with the event predictive model, where a more abstract representation regarding the expected sound in relationship with the set context is stored. The further evaluation process determines in turn whether the current state of the system matches the current goals, this information being once again passed towards and stored into the higher order generative model until the system in engaged into the next (action-) sound processing.

Note that our vision regarding the origin of the auditory intention-based predictions aligns with the idea of ideomotor learning. The proposed model is furthermore congruent with the theory of event coding (TEC), which postulates bidirectional associations between the motor and the event codes (Hommel, 2019; Hommel et al., 2001), as displayed in Fig. 4, in grey, dashed lines. Yet the proposed model additionally allows explaining how the action-based predictions may be reorganized or updated—this is of most importance, since as underlined earlier, the ideomotor framework comes short at explaining the mechanisms of adjusting the action-effect expectations according to new evidence and/or experiences. In this vein, the extended AERS proposes that additional processing steps may mediate the forward and backward connections between the motor system and the auditory event representation. Of particular relevance here is the fact that the backward connection from the auditory event representation to the motor system may pass through additional comparison and evaluation processes; this is when the event predictive model and in turn the higher order generative model feeding information into the motor system may update. We thus believe that these are necessary steps to be considered when clarifying how action-effect sensory predictions may change, rather than think about the bidirectional associations between the motor and event representations as direct connections with no steps in between (see Fig. 4).

A few particularities of the extended AERS require further consideration. First, the motor system input from the higher order generative model does not suggest the exclusion of the original input from the initial sound analysis, where the basic auditory features of a given sound are detected. What it suggests instead is that the communication between the sensory predictive model and the sensory representation concerns the information received from in the motor system, besides the potential information on environmental regularities; if environmental regularities are concurrently extracted, the corresponding motor and non-motor information integrate (or possibly add up, depending on task scenario). Indeed, we have seen that MMN responses to violations of intended action effects are elicited with or without a global regularity pattern (i.e., based on single top-down intention-based expectations, or based on concurrent bottom-up regularity-based and top-down intention-based expectations; Korka et al., 2019). Moreover, the degree to which intention and regularity contribute to the final output (i.e., the auditory event representation) further depends on the specifics of the experimental task. For instance, we have seen that when environmental regularities are difficult to be encoded based on the mere sound probabilities (i.e., stochastic regularities), additional input based on action–effect associations might become the main information source for the sensory predictive model, leading to a larger MMN response when available (Korka et al., 2021). Conversely, it has been repeatedly shown that simple sound regularities, if available, play important roles for the action–effect predictions (Baess et al., 2008; Dercksen et al., 2020; Korka et al., 2020; SanMiguel, Saupe, et al., 2013; SanMiguel, Widmann, et al., 2013). The extended AERS thus offers a framework to study the independent but also joint effects of top-down predictive information, intention on the one hand, and bottom-up extracted regularity on the other hand.

The second aspect requiring consideration is that the information from the event predictive model and the sensory predictive model can be realized directly (via the auditory event representation), without any bottom-up input. This implies that top-down predictions can be implemented in the absence of any actual sensory input (i.e., no existing auditory sensory representation), for instance before the first sound presentation in a previously learned action-effect sequence. This is also the case when representing multiple and simultaneous action–effect associations (Band et al., 2009; Korka et al., 2019, 2020, 2021; Le Bars et al., 2019), when a new action that is coupled with a new sound is planned, which in turn requires a new rule for the event predictive model. Note that from this perspective, the higher order generative model can be seen as a collection of rules, which can be switched between themselves according to the planned action and correspondingly transmitted top-down to the event and sensory predictive model in turn. By contrast, in the case of regularity-based predictions, the rule typically remains until the sensory predictive model updates by bottom-up means.

Perhaps the most relevant example speaking in favour of a direct connection between the event and sensory levels comes from action-related omission studies. As we have previously seen, representing specific key-press–tone associations for each hand that are occasionally omitted leads to omission-related responses, thus demonstrating the intention-based top-down effects in the absence of any sensory stimulation (Korka et al., 2019). This seems to also be the case when no specific action effects can be predicted (i.e., when an action sound is to be expected, but the sound’s identity is unknown), even though the magnitude of the omission responses is reduced in this situation (Dercksen et al., 2020; Korka et al., 2020). This feature of the extended AERS thus potentially accounts for the different components of intentional action (*what, when, whether*; see Brass & Haggard, 2008), which, as we have seen in previous sections, modulate the auditory sensory processing to different degrees. Moreover, note that this connection between the event and sensory levels (in the absence of actual sensory input) is not exclusive to intentional action, but also applies to other top-down predictions—for instance, based on visual information. Indeed, is has been shown before that preceding visual information with predictive value for the subsequent sound modulates the early auditory sensory responses when the sensory predictive model cannot be directly derived from preceding auditory input (Pieszek et al., 2013; Stuckenberg et al., 2019; Stuckenberg et al., 2021; Widmann et al., 2004). In line with this, it could be argued that the extended AERS extends to visual-auditory predictions too, also since it was demonstrated that, similarly to the motor-auditory domain, the perceptual visual-auditory system is flexible and able to form temporal and identity predictions (van Laarhoven et al., 2017; van Laarhoven et al., 2020). However, some differences between the visual-auditory and auditory intention-based predictions likely still exist, potentially because self-evidence leads to stronger predictions, as supported by data from Dogge, Hofman, et al. (2019) who showed that prediction effects were stronger when participants first learned to rely on motor-auditory instead of visual-auditory cues. Similarly, recent data by Ghio et al. (2021) suggested that when self-performed actions and observed actions were well matched in time, the prediction effect following self-performed actions was larger. Indeed, the ideomotor theory also attributes intention a greater role, in contrast to sensory–sensory relations, in the sense that a sensory–sensory relation might or might not result in predictive processing, while intention always does.

Finally, in the light of the connection between the event and sensory levels through which the action-related expectations can be implemented in the absence of a preexisting sensory representation, it could be argued that the direct connection between the motor system and sensory level becomes redundant. Yet studies that implemented in vivo cellular recordings in mice bring forward strong evidence in favour of direct connectivity between the (secondary) motor cortex and auditory cortex areas (Schneider et al., 2014; Schneider et al., 2018), thus demonstrating that a direct motor–sensory connection represents an important element of the action-perception cycle. Accordingly, we would like to propose that these alternative routes account for the different facets of the action-modulated sensory outcomes, rather than being redundant. In this context, hard-wired action effects may be represented by direct communication between motor and sensory systems, while, as we have seen earlier, more abstract action-based expectations may be implemented through the longer communication pathway, including the higher-order generative model and event predictive level. The direct vs. indirect motor-auditory routes are further congruent with the existence of distinctive efference copy vs. corollary discharge signals (Crapse & Sommer, 2008). While both signals are part of the forward model, recent data indicate that they depend on the motor content specificity (Li et al., 2020), in agreement with the nature of our direct vs. indirect connections. In this context, the efference copy potentially corresponds to the connection between our higher order generative model and the event predictive model, while the direct motor-system to sensory-predictive-model connection is likely congruent with corollary discharge.

Thus far, we have discussed how the proposed model processes the information when sounds are to be expected based on the action choice. Yet, as previously mentioned, ideomotor learning assumes bidirectional associations where the reverse is also true (i.e., specific actions are triggered by the sounds that have been previously paired with those actions). Indeed, this seems to be the case as demonstrated, for instance, by studies looking at motor patterns of brain activation while participants passively listened to musical sequences that they previously learned to produce (Haueisen & Knösche, 2001; Stephan et al., 2018). Thus, a final aspect to consider is the relationship between the extended AERS and the auditory-triggered motor responses. As described earlier, our model is congruent with the idea of bidirectional associations between the motor system and the auditory sensory representation. However, we believe that these connections, especially the backward one (i.e., from the auditory event representation to the motor system) do not necessarily have to be direct as suggested by the original ideomotor framework, but may be mediated by further event comparison and evaluation processes which may allow the model to update. Nevertheless, this remains in line with the idea that the information transmitted to the motor system is stored and that sounds that have previously been coupled with actions may activate the corresponding actions.

To sum up, the proposed extended AERS addresses multiple issues. First and most importantly for the scope of this review, it proposes a detailed account of the mechanisms through which intention and action–effect predictions generate effects in the auditory processing hierarchy that is overall compatible with the ideomotor framework, predictive coding, and forward modelling. Additionally, it explains the distinctions and commonalities between how action–effect predictions and regularity-based predictions are achieved at sensory levels and furthermore, accounts for joint effects of the two. We believe that this common perspective could guide the further research studying the joint or individual mechanisms involved in action- and regularity-based predictions. Finally, the proposed trajectories of information processing in the extended AERS in which the communication between the motor and sensory systems can be realized via alternative routes further explains how action–effect predictions may be based on various types on information, in agreement with the notion that action intention represents a multi-dimensional concept.

### The extended AERS and alternative theoretical frameworks

Besides the prediction mechanism based on forward modelling (described in the “auditory attenuation and the forward models” section), a few alternative frameworks of predictive coding/ideomotor origin have been proposed in explaining the action-related sensory processing. The active inference principle, a framework particularly designed to explain action-related predictions or “the enactivist version of the Bayesian brain,” proposes that action and perception integrate to produce optimal behaviour by reducing surprise (i.e., prediction error) following self-produced effects (Brown et al., 2013; Friston, 2011). That is, the theory postulates that agents who perceive and act need to solve the conflict between the self-produced and the external sensory input, this being achieved by adjusting the precision of the sensory evidence following our own actions (Brown et al., 2013). This precision-adjusting mechanism is taken to explain the typical N1 attenuation studies showing that the brain responses following action-related sounds are attenuated by comparison to externally generated sounds (see “auditory attenuation and the forward models” section). However, as we discussed previously, the N1 attenuation phenomenon is a rather broad sensory effect that cannot clearly be attributed to intentional action; instead, it seems to reflect a comprehensive mechanism that might include but is not limited to intentional action. Furthermore, while the precision adjustment hypothesis is generally used to explain the magnitude of environmental-related sensory predictions (Feldman & Friston, 2010; Friston, 2018), in the context of action-related predictions, it merely states that action-related outcomes are differently weighted relative to the environmental-related outcomes, without addressing the specific role of expected vs. unexpected or specific versus unspecific action effects. From this perspective, the extended AERS offers a more detailed explanation that refers to both the specifics of the action–effect predictions, as well as their relation to possible concurrent predictions based on environmental regularities.

One further model proposed by Verschoor and Hommel (2017) explains how the ideomotor principle and predictive coding might integrate in the context of action. Concretely, they propose that the anticipation of the intended effect activates the respective effect representation, which in turn cues action selection, as the original ideomotor principle postulates. Then, the activated representations are used to generate a prediction regarding the action outcome which, in accordance with the predictive coding theory, is evaluated against the actual sensory feedback; in case the two are incompatible, prediction error is transmitted back up the cortical hierarchy in order to update the generate model. Thus, from this perspective, the model can be described in two steps: ideomotor *action selection*, followed by prediction error-based *action evaluation*. While this perspective provides an elegant theoretical connection between the two theories, note that the focus falls onto explaining the action-related outcomes. By contrast, the extended AERS focuses on explaining the sensory outcomes, as modulated by the action–effect predictions. This is an important distinction, as in the first case, the evaluation of action-related outcomes taps into the opposite direction of the perception–action cycle by focusing on the specifics on the motor rather than the sensory event representations.

Finally, compared with both the active inference principle (Brown et al., 2013) as well as the model of Verschoor and Hommel (2017) that are rather domain-general, the extended AERS is specific to the auditory system. A level of general cross-modality conceptualization of the predictive mechanisms is crucial for an overall understanding of the brain’s fundamental functioning. Yet the details of the modality-specific mechanisms are similarly important if we consider that the value of predictive processing might be larger in the context of more transient information (as is the case in audition; see also Winkler & Schröger, 2015) compared with information that we can revisit more easily (as in the case in vision).

## Summary

The present work brought forward the role of action intention-based predictions on the processing of action-related auditory outcomes. We reviewed relevant studies with a focus on recent results looking at action–effect predictions from three main lines of research (without limiting ourselves to these). First, the sensory attenuation studies brought evidence that, overall, the attenuation phenomenon presumably representing forward modelling is not limited or specific to intentional action, as often described; intention might nevertheless contribute, if/when available. Second, in line with the ideomotor theory, suggesting that actions are encoded together with their sensory outcomes, studies including active oddball paradigms demonstrated that various steps in the auditory processing hierarchy are differentially modulated depending on whether the produced sounds matched or mismatched the expected action affects. Third, studies employing omission paradigms demonstrated the existence of specific vs. unspecific action–effect predictions along the auditory processing hierarchy, in line with the view that intentional action represents a multidimensional concept, which may refer to the identity or the timing of the expected action-effect. Integrating findings from all reviewed paradigms, we proposed a theoretical model—the extended auditory event representation system (AERS)—to explain the mechanisms through which intention-based predictions modulate the auditory processing hierarchy, and additionally, to explain the distinctions and commonalities between intention- and sensory regularity-based predictions. We believe that this common perspective could guide further research studying the joint or individual effects of the two prediction types at auditory sensory levels.

## Footnotes

1 In this system, one could be interested to zoom in on the specifics of each module, in a process of hierarchical decomposition; in this sense, the model may never be considered complete. Here, the individual modules should be regarded as different levels of abstraction and complexity, rather than describing full-scale processes.
